# A building height dataset across China in 2017 estimated by the spatially-informed approach

**DOI:** 10.1038/s41597-022-01192-x

**Published:** 2022-03-11

**Authors:** Chen Yang, Shuqing Zhao

**Affiliations:** College of Urban and Environmental Sciences, and Key Laboratory for Earth Surface Processes of the Ministry of Education, Peking University, Beijing, 100871 China

**Keywords:** Sustainability, Geography

## Abstract

As a fundamental aspect of the urban form, building height is a key attribute for reflecting human activities and human-environment interactions in the urban context. However, openly accessible building height maps covering the whole China remain sorely limited, particularly for spatially informed data. Here we developed a 1 km × 1 km resolution building height dataset across China in 2017 using Spatially-informed Gaussian process regression (Si-GPR) and open-access Sentinel-1 data. Building height estimation was performed using the spatially-explicit Gaussian process regression (GPR) in 39 major Chinese cities where the spatially explicit and robust cadastral data are available and the spatially-implicit GPR for the remaining 304 cities, respectively. The cross-validation results indicated that the proposed Si-GPR model overall achieved considerable estimation accuracy (R^2^ = 0.81, RMSE = 4.22 m) across the entire country. Because of the implementation of local modelling, the spatially-explicit GPR outperformed (R^2^ = 0.89, RMSE = 2.82 m) the spatially-implicit GPR (R^2^ = 0.72, RMSE = 6.46 m) for all low-rise, mid-rise, and high-rise buildings. This dataset, with extensive-coverage and high-accuracy, can support further studies on the characteristics, causes, and consequences of urbanization.

## Abstract (summary)

**Table:** 

Measurement(s)	1 km gridded building height across China in 2017
Technology Type(s)	Sentinel-1 SAR; Spatially-informed Gaussian Process Regression
Factor Type(s)	Sentinel-1 SAR
Sample Characteristic - Location	China

## Background & Summary

In this century of the city, urban growth is sweeping the planet powerfully and irreversibly^1^. Globally, people living in cities have outnumbered rural dwellers. As of 2018, cities housed 55% of the world’s population, up from roughly 30% in 1950 and expected to rise to 68% by 2050^2^. Cities are hubs of population and capital, crossroads of civilizations, and catalysts for innovation. Cities, on the other hand, are hotbeds for crime, deprivation, and disease^3^. Consequently, urbanization creates enormous opportunities for social and economic progress, whereas at the same time, brings with it a slew of global challenges, such as climate change^4,5^, resources access^6,7^, and public health^8^.

Urban form is a critical component of the nexus between urban growth and sustainable development^9^. There is strong evidence that urban form, as well as its associated urban density and functions, has a significant impact on resource/material consumption and even climate change^10,11^. Thanks to the proliferation of earth observation (EO) data and the boom of various open data, the scientific community has been able to characterize the horizontal morphology and evolution patterns of urban areas (human settlements) at multiple scales^12–16^. Despite the plethora of new insights into horizontal urban forms, such knowledge is increasingly under-powered in supporting urban transformations towards sustainability^9,12,17^. Using impervious surfaces as a spatial proxy of cities not only reflects a quite limited proportion of built-up spaces, but it also ignores the land-use intensity within urban areas^18,19^. Therefore, it is imperative to do spatially-explicit building height estimation. The consideration of vertical dimensions will provide fresh insights into urban landscape structures and urbanization pathways, as well as the ability to project future emissions under urbanization and climate change.

The increasing availability of EO data has allowed building heights to be estimated on a broad spatial scale. Synthesized aperture radar (SAR) data, such as Sentinel-1, has been the primary data source in three-dimensional urban morphology characterization for its ideal balance between spatial coverage and resolution^20–22^. Machine learning approaches are increasingly being used to establish the relationship between building height and SAR backscatter, and there were pioneer efforts devoted to such work. For example, Li, *et al*.^23^, using Sentinel-1 SAR data and auxiliary data, developed a Random Forest model to map 3D building structures over China, Europe, and the United States. Frantz, *et al*.^24^ estimated building heights across Germany based on Support Vector Regression and Sentinel-1 and Sentinel-2 time-series. Nonetheless, most existing studies estimate building height based on aspatial regressions, which makes it difficult to reflect the spatially non-stationary association between SAR backscatter and building height^22^. The incorporation of spatial information has proven to be a significant complement to existing aspatial regressions, whether for building height estimation^24^ or other modeling, such as urban climate^25–27^. This paper demonstrates an attempt to bridge the above knowledge gap by incorporating spatially-explicit/implicit information into a machine learning model.

As a rising powerful economy with nearly the fastest urban expansion, China’s urbanization is critical to its own sustainability and that of the world^28^. In the current developmental context, urbanization is being emphasized as a major fuel for expanding the domestic demand^28,29^. To contribute to global sustainability and to facilitate ongoing urbanization, China announced the ambitious urbanization plan in 2014. The plan is not only China’s first nationwide coordination of urbanization, but also a long-term sustainable response to the past’s crude urbanization^30^. Furthermore, the Chinese government pledged that national CO_2_ emissions would peak by 2030. Given the crucial role of building height in accounting urban material flows and carbon emissions, a wall-to-wall building height map in China would strongly support Chinese efforts towards global sustainability as well as its domestic development. To date, building height mapping efforts have been emerging in China from specific cities^31^ to regional^32^ and even national scales. Ren *et al*.^31^ used the digital surface model to obtain building heights within Hong Kong. Li *et al*.^32^ generated a building height map covering 36 Chinese cities from the web map. Li *et al*.^23^ estimated building heights across mainland China in 2015 using Sentinel-1 SAR data. Building height data covering the whole China, however, is still limited, particularly with consideration of spatially informed data.

Thus, the goal of this study is to use the spatially-informed Gaussian process regression (Si-GPR) to create a wall-to-wall 1 km resolution building height dataset across China in 2017. For validation, the estimated building height was compared to cadastral references and reliable open data.

## Methods

Since China demonstrates diverse built-up landscapes due to its transient but dramatic urbanization, our aim is to facilitate the characterization of vertical urban landscapes in China and to fill current building height data gaps in China. Our research results are expected to support further studies on the characteristics, causes, and consequences of urbanization, particularly material flux accounting and emissions reduction efforts in China. Besides, the methodology for estimating building heights using space-borne SAR data and machine learning methods can be transferred to other regions, particularly the Global South.

### Data collection and pre-processing

In the estimation of building heights, we primarily used (1) Sentinel-1 SAR Ground Range Detected (GRD) time-series^33^, (2) biophysical indices [including normalized difference vegetation index (NDVI)^34^, normalized difference built index (NDBI)^35^, and surface albedo^36^] derived from Sentinel-2 surface reflectance (SR) imageries^37^, (3) Visible Infrared Imaging Radiometer Nighttime Day/Night Band Composites (VIIRS DNB nighttime light)^38^, (4) global annual impervious area (GAIA) maps by Gong, *et al*.^39^, and (5) building references. The collection and preprocessing of Sentinel-1 data, biophysical indices, VIIRS DNB nighttime light (NTL), and GAIA maps were conducted on the Google Earth Engine platform. The filtering and collating of reference building data were performed with the ESRI ArcGIS Pro 2.5 software. The entire set of data was reprojected into the Albers Conical Equal Area coordinate system.

We first generated the 1 km × 1 km fishnet over China using the same projection coordinate system with the above datasets. Using the GAIA subset in 2017, the imperviousness of each 1 km × 1 km cell was then calculated, and only cells with impervious surface coverage greater than or equal to 25% were identified as built-up cells for subsequent height estimation. Furthermore, the coverage of impervious surfaces within each cell is treated as an independent variable in the estimation.

Under the Aggregation-then-Prediction strategy, Sentinel-1 and Sentinel-2 time-series for the 2017 winter season (Dec 1^st^ 2016 - Mar 31^th^ 2017 and Dec 1^st^ 2017 - Mar 31^th^ 2018) were obtained in order to minimize the uncertainty in building height estimation caused by unman-made vertical landscapes (e.g., vegetations) inside the built-up environment. In the estimation, we used the annual median of the above time-series as explanatory variables for the modeling, aiming at accounting for critical seasonal dynamics. We obtained the monthly average NTL intensity for the 12 months of 2017 for VIIRS NTL data to approximate typical patterns of socioeconomic activity throughout the year. The Sentinel-2 derived biophysical indices were calculated using the annual median Sentinel-2 surface reflectance. To eliminate bias from building overlay and satellite side-views, Sentinel-1 backscatter for two polarizations (VV and VH) and two orbits (ascending and descending) were incorporated in the estimation.

The use of Sentinel-2-derived biophysical indices has the potential to reduce the ambiguity in the relationship between SAR backscatter and building height. To correct unexpected ground roughness recorded in SAR data caused by non-man-made vertical objects, NDVI was introduced as an independent variable. NDBI, in collaboration with VIIRS NTL, reflects the built-up environment’s building structures and land-use intensity. Surface albedo can represent building materials, which is informative for estimation performance. We spatially averaged the values within the built-up environment into 1 km × 1 km built-up cells. The vectorized GAIA data was adopted to impose a spatial constraint on the built-up environment, masking out values that were outside of it.

There are two primary sources of reference building data in the estimation: (1) cadastral data provided by local authorities, and (2) open building data obtained from web maps [i.e., Amap (https://www.amap.com/) in this study]. We primarily used cadastral data as references in the estimation because, on the one hand, of its considerable data completeness and accuracy. And, on the other hand, it covered most of China’s major cities, which have a more diverse vertical urban landscape (i.e., a wide variety of building heights inside these cities) than other cities. In Shanghai, for example, a considerable number of built-up cells (over 30.27%) are taller than 40 m, while the majority of built-up cells (54.04%) are dominated by low-/medium-rise buildings (less than 24 m). Thus, the cadastral data of the 39 major cities overall ensures a wide range of building heights in the reference sample, maximizing the performance of machine learning regression^24^. The building height estimation effectiveness of Si-GPR is expected to improve further (e.g., minimizing potential overestimation due to the limited coverage of cadastral data) as the reference sample is expanded outside the current 39 cities. The cadastral data, in general, covered the downtowns of 39 major Chinese cities, including four municipalities, twenty-eight provincial capitals, five sub-provincial cities, and Hong Kong and Macau. Open building data is also important, not only as a supplement to cadastral data, but also as a cross-validation between the two. The scattered buildings have been removed from the reference building data, and the open building data has been visually interpreted to calibrate the heights. Moreover, outliers in the reference data were also identified and eliminated, including buildings that were either extremely huge (>20,000 m^2^) or too tiny (<50 m^2^) to be permanent structures^40^. Chimneys and water towers, for example, were among the constructions that topped 100 meters but had a footprint of only a few dozen square meters of floor space. These buildings (about 106 buildings in total) were eliminated during the visual calibration because they obviously exhibited egregious height errors and area mistakes compared to other nearby buildings. Finally, 327,649 buildings were acquired as reference data, with 211,596 buildings (~64.57%) recorded in cadastral data and the remaining 116,053 buildings (~35.43%) contributed by open data. These reference buildings are distributed across 48,365 built-up cells in China, accounting for approximately 13.57% of all built-up cells (356,185 built-up cells in total).

### General framework

**Fig. 1:**
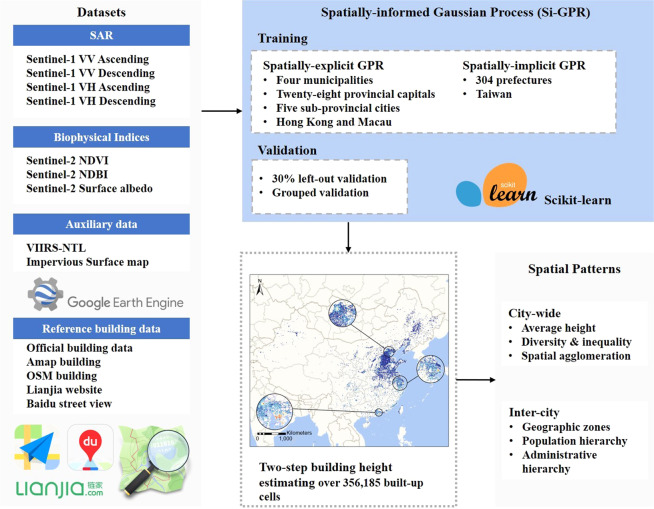
The proposed framework of estimating building heights using the Spatially-informed Gaussian process regression (Si-GPR) in China.

We estimated building heights over 356,185 built-up cells across China in 2017 based on Sentinel-1 SAR backscatter using the framework in Fig. 1. The proposed framework contains three major steps. First, we conducted data collection and preprocessing on the Google Earth Engine platform, ArcGIS Pro 2.5 software, and web-maps (including Amap and Baidu Map). Second, we established the Si-GPR model based on the Sci-kit Learn machine learning package in Python^41^. The Si-GPR model is made up of two parts: a spatially explicit GPR and a spatially implicit GPR that estimate building heights for the 39 main cities and the other 304 prefecture-level cities, respectively. As a result, Si-GPR training is divided into two distinct parts. A 30 percent left-out cross-validation was used to test the Si-GPR model’s estimation performance, and further group validation was undertaken for buildings of various heights (i.e., low-, mid-, and high-rise buildings). Third, we employed a two-step strategy to estimate building heights over 356,185 built-up cells across China. For the city-by-city estimate of 39 large cities and the one-batch estimation of the remaining 304 cities, spatially-explicit GPR and spatially-implicit GPR were utilized, respectively. Besides, we summarized the spatial patterns of building height distribution from the city and inter-city perspectives.

### The spatially-informed Gaussian process regression (Si-GPR) model

As introduced above, the Si-GPR is incorporated by a spatially-explicit Gaussian process regression and a spatially-implicit Gaussian process regression. Although the spatially explicit Gaussian process regression is a reliable and effective solution for estimating building heights, it is not suitable for nation-scale mapping. First, the accessibility of samples limits the broad-scale deployment of the spatially-explicit Gaussian process regression. It is generally known that spatially-explicit models rely on well-distributed and reliable spatial samples^42^, which are not readily available in a country the size of China. Second, modeling and calibration of spatially explicit Gaussian process models are time-consuming and computationally resource-intensive, making estimation with spatially-explicit Gaussian process models over 300+ cities problematic. The accuracy of building height estimation using global regression has been demonstrated to be satisfactory at the national-scale^23,24^. And the accuracy is expected to improve when spatial correlations are implicitly included.

The spatially-implicit Gaussian process regression is a global regression, which means that all samples are trained collectively in the model, and then building height is estimated in all built-up cells in a batch. The spatial relationships in the spatially implicit Gaussian process regression are implied by the spatial projection coordinates of the built-up cell as well as the city code where the cell is located. The general representation can be depicted as shown in Eq. 1.

1 $$BH={f}_{GPR}(SAR,NDVI,NDBI,Albedo,ISF,NTL,Coord,City\_ID)$$

Where *BH* denotes the building height of a built-up cell, *ISF* is the impervious surface fraction within the cell, *Coord* and *City*_*ID* are the projection coordinates of the cell and the identification code of the city where it is located, which is an intra-city and inter-city position metric, respectively. *City*_*ID* is a six-digit code that is unique to each Chinese city (e.g., 110100 for Beijing) and is unified by taking into account the geographic context, socioeconomic development, and administrative affiliation of a specific city.

In the estimation, the spatially-explicit Gaussian process regression is run city-by-city based on city-wise sample sets. The spatially-explicit Gaussian process regression creates a spatially different local regressor for each cell to be estimated. Samples within a particular distance (denoted as bandwidth here) from a specific to-be-estimated cell are believed to be the most explanatory in each local regressor. To establish the correlations between the building height and independent variables, only these neighboring samples will be used. The bandwidth of the spatially-explicit Gaussian process model is determined by iterative cross-validation, similar to the geographically weighted regression (GWR). The spatially-explicit Gaussian process model, however, does not compute the geographically weighted average of the estimates for each local regressor, unlike GWR. For each built-up cell to be estimated, a local Gaussian process regressor can be mathematically represented as:

2 $$B{H}_{i}={f}_{GPR\left({u}_{i},{v}_{i}\right)}(V{V}_{i},V{H}_{i},NDV{I}_{i},NDB{I}_{i},Albed{o}_{i},IS{F}_{i},NT{L}_{i})$$

**Fig. 2:**
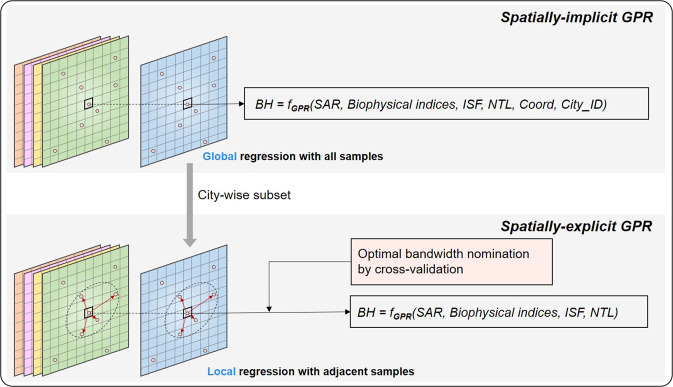
Schematic of the Si-GPR model for building height estimation.

Where *BH*_*i*_ denotes the estimated building height of cell *i*, which is located at (*u*_*i*_, *v*_*i*_). *f*_*GPR*(*ui*,*vi*)_(·) represents the location-specific Gaussian process regressor. Specifically, both the global regressor and the local regressor in the Si-GPR model are constructed and calibrated using the Gaussian process regression function in sci-kit learn package^41,43^. Figure 2 shows the schematic of the Si-GPR model for building height estimation across China.

## Data Records

**Fig. 3:**
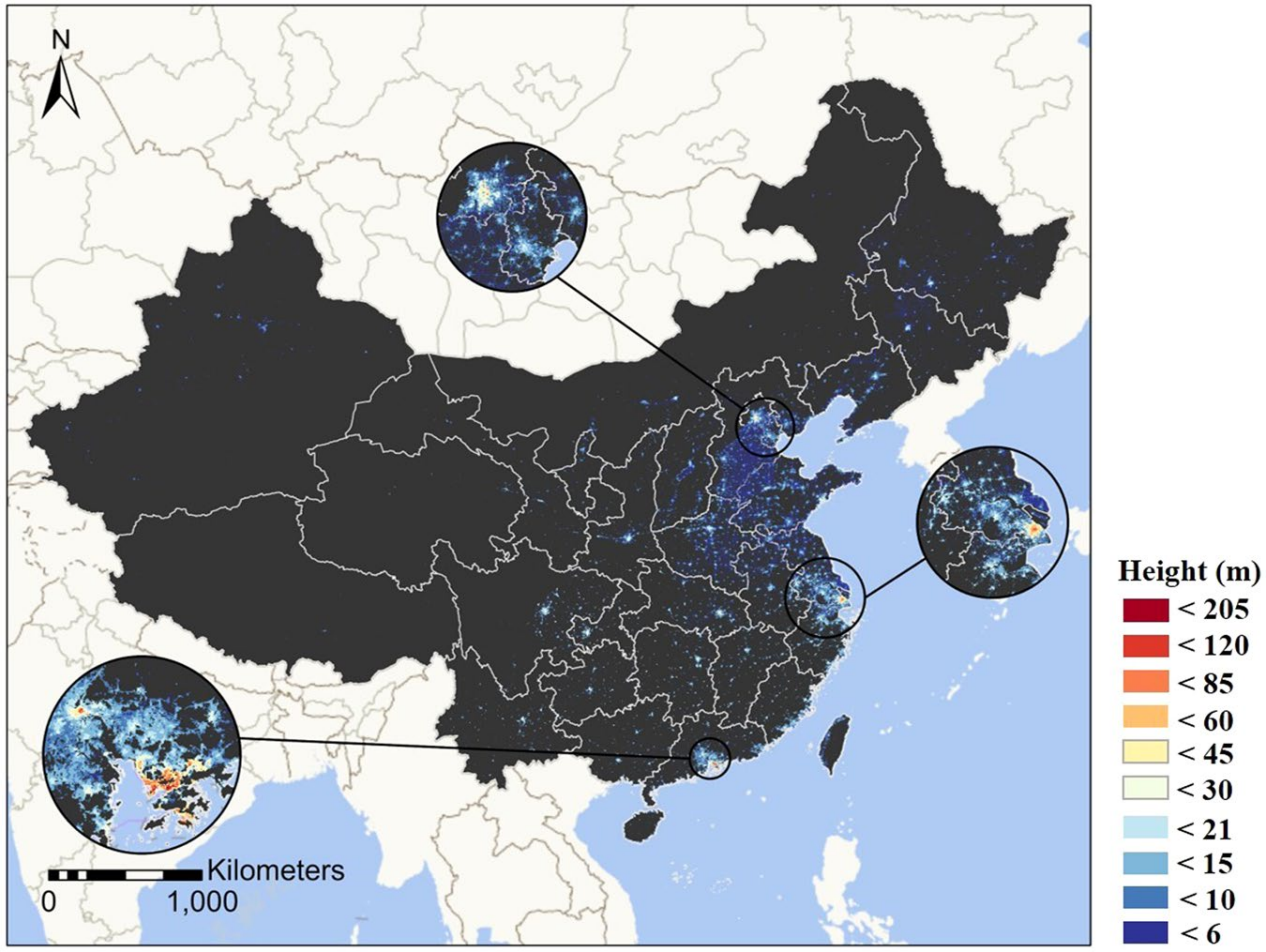
The spatial pattern of building heights across China in 2017.

Figure 3 depicts the estimated wall-to-wall building height dataset for China in 2017. The building height dataset is tagged in GeoTIFF file format at the 1 km × 1 km spatial resolution in the Albers Conical Equal Area coordinate system. The value of a grid represents the average height of artificial built-up structures within it, including roads and parking lots, etc. This dataset was estimated on a Lenovo DeepComp-X8810 supercomputer (2*Intel Xeon Gold 6142, 2.6 GHz, 128 GB) from the High-performance Computing Platform of Peking University. This dataset can be visualized and processed by GIS software (e.g., QGIS and ESRI ArcGIS). The building height dataset has been made public under Figshare (10.6084/m9.figshare.14999067.v2)^44^.

## Technical Validation

### Estimation performance assessment

**Fig. 4:**
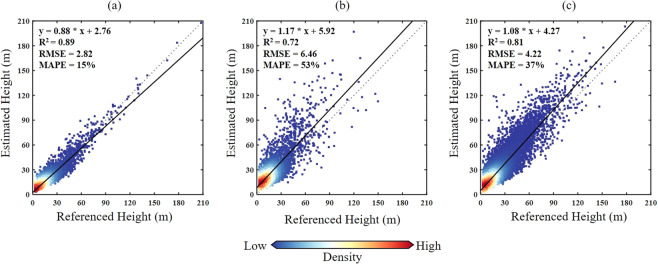
Building height validation on 30% left-out samples of the spatial-explicit Gaussian process regression (**a**), the spatial-implicit Gaussian process regression (**b**), and the Spatial-informed Gaussian process regression (**c**).

Three 30% left-out cross-validations were undertaken independently to evaluate the performance of the spatially-explicit GPR, the spatially-implicit GPR, and the combined Si-GPR model in building height estimation. R-square (R^2^), root mean squared error (RMSE), and mean absolute percentage error (MAPE) were three measures used to evaluate estimating effectiveness qualitatively. The estimation accuracies of spatially-explicit and spatially-implicit GPR were assessed using 30% of the reference sets within their operational geographic extents (i.e., 39 major cities and the remaining 304 prefecture-level cities), respectively. Following that, we randomly selected 14,510 built-up cells (~33.07% of total samples) from the reference set to serve as the validation set for the performance evaluation of the Si-GPR model. In other words, the remaining 33,855 built-up cells have been partitioned into training sets for the spatially-explicit and spatially-implicit models according to the cities they are located in. The estimated-versus-observed plots in Fig. 4 reveal that Si-GPR can estimate building heights in China with reasonable accuracy (R^2^ = 0.81, RMSE = 4.22 m). Not unexpectedly, the estimation performance of the spatially-explicit GPR (MAPE = 15%) outperformed the spatially-implicit GPR (MAPE = 53%). The linear-fitted slopes of estimated-observed relationships show that the spatially implicit model overestimates building height (slope = 1.17) while the spatially explicit model slightly underestimates (slope = 0.88) it. Despite an overall underestimation of building height, the spatially-explicit model can estimate extreme-high buildings (>100 m) with great accuracy, as illustrated in Fig. 4. This should be thanks to the intrinsic local modeling of the spatially-explicit Gaussian process regression. Besides, there was random building height overestimation and underestimation in the estimated height by the spatially-implicit Gaussian process regression (and the consequent Si-GPR). On the one hand, substantial over-/under-estimation can be attributed to issues such as backscatter uncertainties caused by complex building structures and building material diversity, as well as errors in reference buildings. On the other hand, the randomness of such estimating mistakes suggests that there should be no systematic bias in the spatially-implicit GPR’s implementation.

**Fig. 5:**
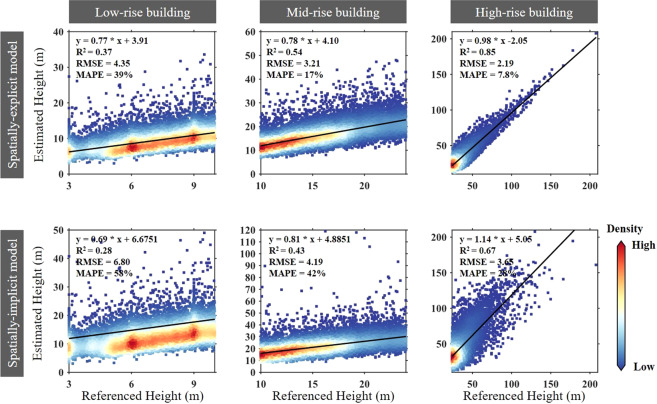
Scatterplots of the reference height and estimated height for low-rise buildings (3–9 m), mid-rise buildings (10–24 m), and high-rise buildings (>24 m).

Furthermore, a grouped cross-validation was designed to collect the same amount of samples for low-rise (3–9 m), mid-rise (10–24 m), and high-rise buildings (>24 m), allowing for a more thorough examination of the model’s estimation for multiple building height hierarchies. As shown in Fig. 5, both the spatially-explicit model (RMSE reduced by 2.16 m) and the spatially-implicit model (RMSE reduced by 3.15 m) both performed better when estimating the heights of high-rise buildings. It can be seen that introducing the local modeling technique improved the Gaussian process regression estimation performance, especially in built-up cells dominated by high-rise buildings. However, regardless of whether the model is spatially explicit (MAPE = 39%) or spatially implicit (MAPE = 58%), obtaining satisfactory estimation accuracy over built-up cells dominated by low-rise buildings is always problematic. One possible explanation can be given here that high-rise buildings tend to be located in well-planned modern neighborhoods, while low-rise buildings are often located in less-developed urban regions, such as urbanized villages^28,45^. As a result, low-rise buildings in China generally lack standardized building norms and have various building materials, resulting in more complex SAR backscattering features and consequently uncertainties in the BH-SAR correlations for low-rise buildings^20,22^.

### Comparison with existing studies

Great advances have been made in estimating building height from SAR data but generally based on aspatial regressions^22–24^. Li *et al*.^22^ developed an indicator-based model by combining the Sentinel-1 VV and VH backscatter. This model is concise and effective since it achieves acceptable estimation accuracy (RMSE = 1.5 m, MAPE = 44%) without the need for a large number of reference samples or time-consuming preprocessing. To map the fine-resolution (~10 m) building height in German, Frantz *et al*.^24^ coupled Sentinel-2 multispectral imageries with Sentinel-1 SAR backscatter data in a SVR regression. Frantz *et al*.^24^ selected 50 features, including six biophysical indices derived from Sentinel-2 imageries and spatial information obtained using morphological approaches, to improve the building height mapping accuracy. In addition, the rigorous and meticulous reference sample filtering in their study also contributed to the ideal estimation performance of SVR (RMSE ≈2.92 m). Furthermore, Li *et al*.^23^ used a random forest model combined with a diverse explanatory sample of spectral reflectance, biophysical indicators, NTL, and socioeconomic factors (e.g., roads and urban footprint) to implement inter-continental building height estimations across China, the United States, and Europe in 2015. In this study, we created a wall-to-wall 1 km resolution building height dataset across China in 2017 using the spatially-informed Gaussian process regression.

**Fig. 6:**
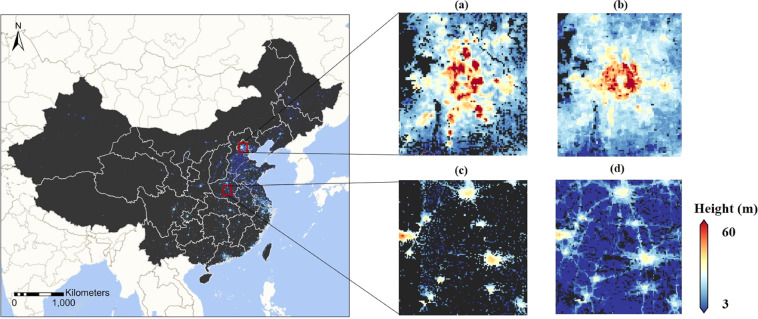
Inter-comparison of building height estimation in this study (**a**,**c**) and by Li *et al*.^23^ (**b**,**d**).

We further inter-compared our estimated map with the building height map in 2015 generated by Li *et al*.^23^. However, owing to a lack of validation data throughout the two study periods, we forewent quantitatively evaluating the estimated performance of the two maps and instead conducted a visual comparison of the building height patterns reported by them. As shown in Fig. 6, the building height map in this study demonstrated more null values than the map by Li *et al*.^23^ This was actually due to the differences in the built-up mask and not related to the building height estimation performance^24^. The building height map of this study differed greatly from that of Li *et al*.^23^ within Beijing (Fig. 6(a,b)). Their map well reflected the standing of taller buildings around the ring roads and the fact that the buildings surrounding the Forbidden City were relatively lower. In contrast, our results highlighted the dominance of key regions within Beijing in the vertical landscape, which were either main business districts or high-tech parks. In fact, the differences between the approaches used to estimate building heights within Beijing for the two studies, that is, site-based spatially-explicit GPR in this study and the global RF in Li *et al*.^23^, may be the primary reason for explaining the difference in the spatial pattern of building heights between the two maps. It can also be related to the fact that urbanization including vertical expansion can be very dramatic in a metacity like Beijing as evidenced by vertical expansion only captured in our study in the regions prioritized as regional sub-centers in the municipal planning of Beijing, so it was reasonable that more vertical expansion occurred within such regions in 2015–2017 than in other regions. And the building height patterns shown by these two building height maps were quite similar in several cities located in central China (Fig. 6(c,d)). The heights estimated in this study are slightly higher than those estimated by Li *et al*.^23^, which could be attributed to the vertical growth in these cities in 2015–2017. Such a resemblance demonstrated that the spatially-implicit GPR achieved comparable estimation accuracy to RF in the areas with relatively simple landscape features once spatial information has been involved.

Generally, the advantages of the proposed Si-GPR over the existing methods are twofold: firstly, the resulting building height maps have more spatial details thanks to the spatially explicit modeling approach; and secondly, the incorporation of spatial information and spatial relationships allows Si-GPR to achieve estimation accuracy comparable to that of the global model with fewer explanatory features. However, model implementation and calibration of spatially explicit GPR are more time-consuming than existing global regressions.

### Limitations and future work

Benefited from increasingly advanced earth-observation and high-performance computation techniques, we are able to investigate three-dimensional urban landscapes at a broad scale. However, like many other published efforts, the building height map across China produced by this work relies on data that has only recently been available, making the generation of building structures time-series challenging. Besides, the qualities of many machine-learning models (including Gaussian process regression) follow the ‘garbage in, garbage out’ principle^46^, i.e. the ideal estimation accuracy of the Si-GPR model is still based on high-quality reference building datasets. Most countries and regions, particularly those in the Global South, lack access to high-quality cadastral data and open web-map data.

Therefore, in future works, we will focus our efforts on two aspects: first, we will explore the associations between building structures and globally-covered proxies (e.g., NTL) to enable time-series estimation of worldwide building height estimation. And more advanced artificial intelligence (AI) techniques (e.g., deep learning^47^) are expected to lessen the reliance of estimation performance on training set quality.

## Usage Notes

**Fig. 7:**
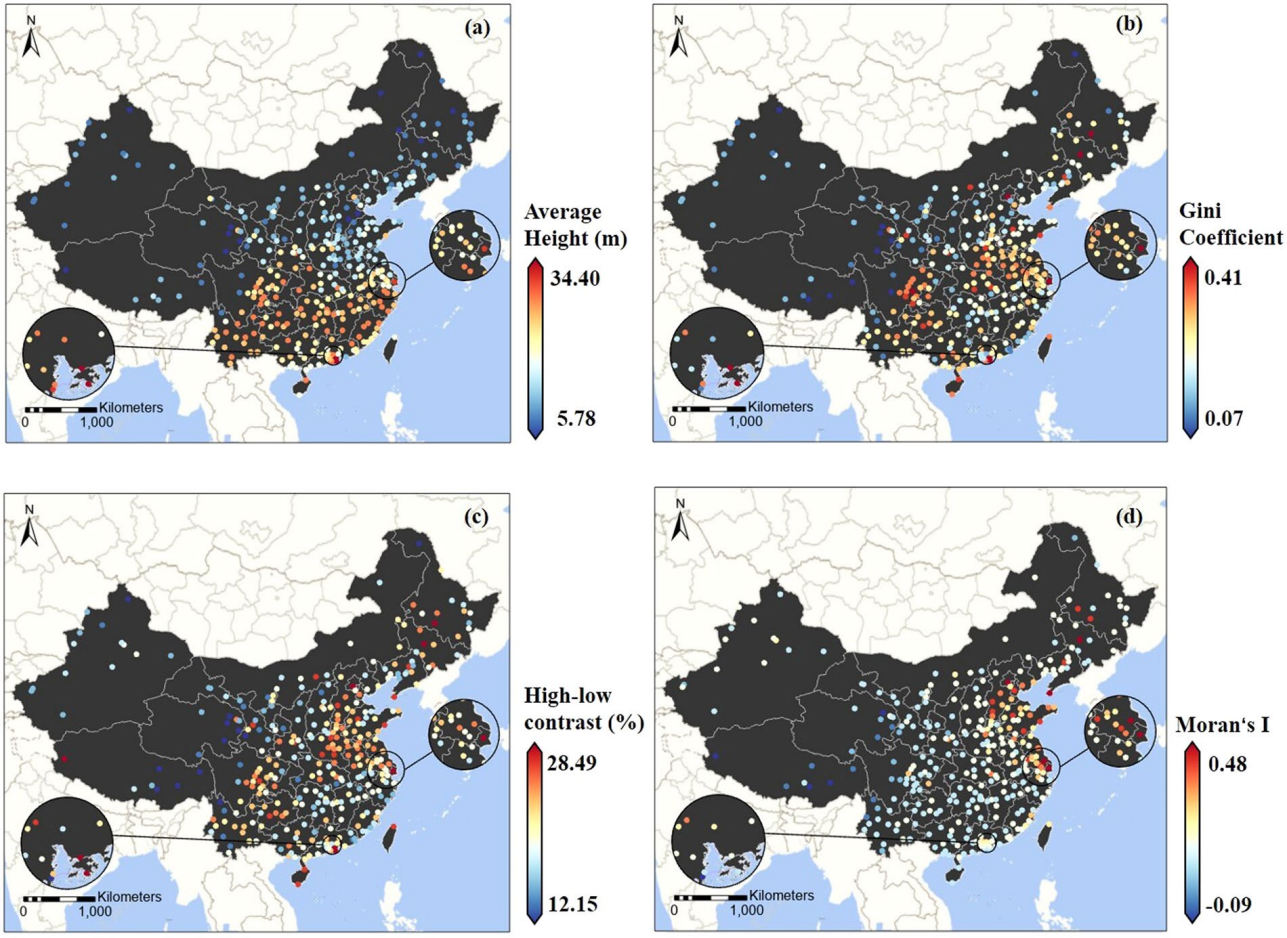
Spatial patterns of building height with the cities, measured by the average height (**a**), Gini coefficient (**b**), high-low contrast (**c**), and Moran’s I (**d**).

**Fig. 8:**
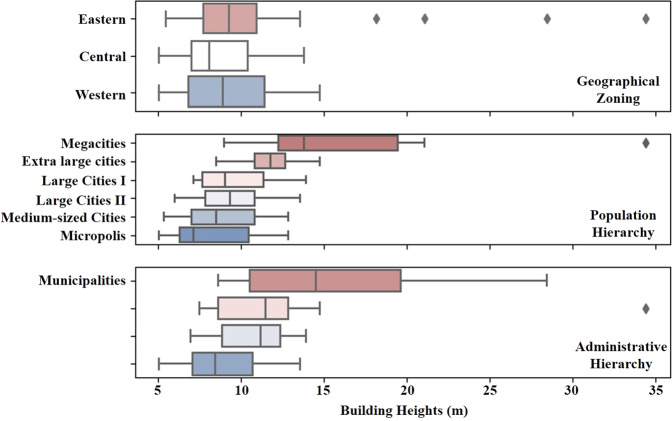
Cities’ average heights for various geographic zones and population/administrative hierarchies.

The estimated building height dataset will be a valuable product supporting applications that require extensive-coverage and high-accuracy information on vertical urban landscapes across China. This dataset can help fill the existing data gaps and will enable derivative measurements on built-up structures for both the scientific and social communities. To support the usage of the dataset, we provide an example to illustrate the spatial disparities of building height across China. First, we adopted four metrics (i.e., average height, Gini coefficient, high-low contrast, and Moran’s I) to investigate the building height patterns from the city perspective. The Gini coefficient, for example, measures the inequality of building height distribution within a city, and the high-low contrast, which uses the sum of the heights of the top 10% of cells as a percentage of the city’s total, quantifies the dominance degree of taller buildings in the city’s vertical urban landscapes. The average heights of southern Chinese cities are higher than those of northern cities, as illustrated in Fig. 7, whereas the agglomeration of taller buildings within eastern coastal cities is greater. Taller structures dominate the vertical landscapes of cities in China’s more urbanized regions (such as the Jianghuai plain, North China Plain, and Chengdu plain), resulting in larger differences in building heights (higher Gini coefficients). We can deduce from the intra-city perspective (Fig. 8) that population agglomerations and administrative capacity are the primary determinants shaping the diverse vertical built-up structures, while differences in geographic zoning do not result in such significant variances in average building heights.

**Fig. 9:**
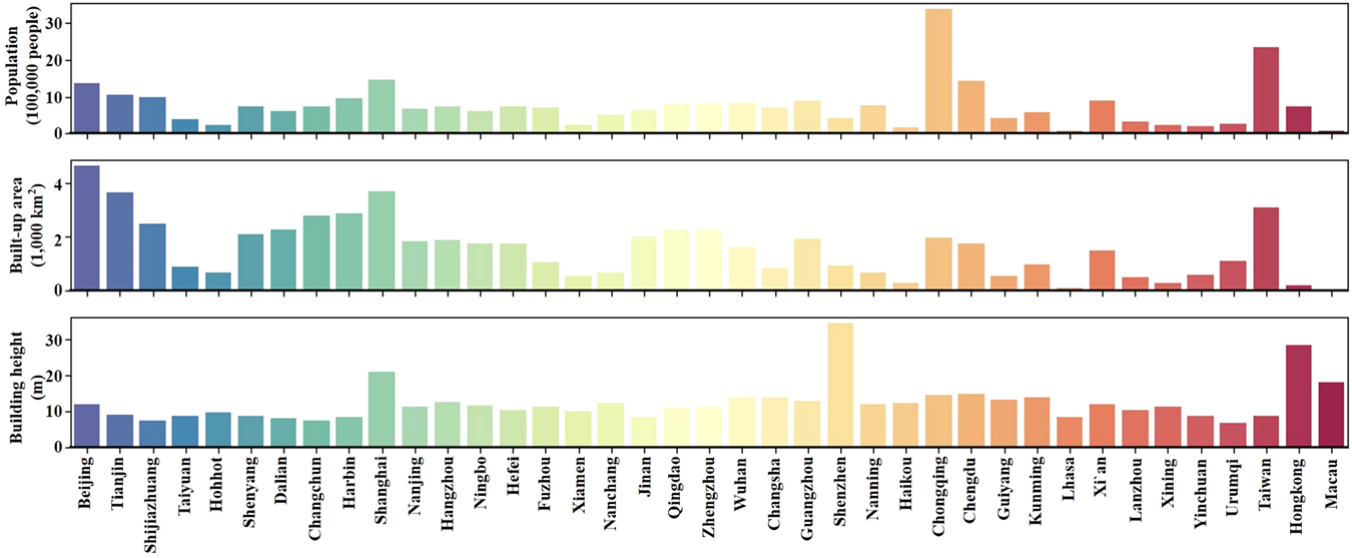
Populations, built-up areas, and average building heights for 39 major Chinese cities.

Furthermore, building height, as an additional measurement of built-up spaces, will enable us to examine the urban scale through a new lens. As indicated in Fig. 9, the traditional size rank of the city system based on population or the horizontal built-up area will be disrupted by the vertical perspective of built-up structures. Further assessments of city coevolution and relative characteristics in both space and time could benefit from such a new measurement of city scale^29,40,48,49^.

Besides, urban form has been identified as an important factor in determining urban carbon emissions and energy consumption^11^. As one of the fundamental measures of urban form, nation-scale mapping of building heights is expected to contribute to the evaluation of carbon budgets and energy consumption efficiencies in Chinese cities and further support China’s ambitious plan for peaking carbon dioxide emissions before 2030^50,51^. This dataset, incorporated with other earth observation data and non-earth observation data, can also be used to support studies concerning human wellbeing, such as climate change^4,52^, social inequalities^53,54^, public health^55^.

## Data Availability

The programs used to generate all the results were Python, Google Earth Engine (GEE) and ESRI ArcGIS (Pro 2.5). The scripts of data collection and preprocessing on GEE can be accessed on GitHub (https://github.com/terryyangwhu/BH_China.git). Furthermore, we have made the Si-GPR model’s source code publicly accessible on GitHub (https://github.com/terryyangwhu/BH_China.git).
